# Mortality and disability risk among older adults unable to complete grip strength and physical performance tests: a population-based cohort study from China

**DOI:** 10.1186/s12889-024-18258-7

**Published:** 2024-03-13

**Authors:** Yu Cheng Huang, Ying Dong, Chen Ming Tang, Ying Shi, Jian Pang

**Affiliations:** 1 Shi’s Center of Orthopedics and Traumatology, Shuguang Hospital, Shanghai University of Traditional Chinese Medicine, 201220 Shanghai, China; 2 Institute of Traumatology & Orthopedics, Shanghai Academy of Traditional Chinese Medicine, 201220 Shanghai, China; 3 School of Public Health, Shanghai University of Traditional Chinese Medicine, Shanghai, China; 4 Shuguang Hospital, Shanghai University of Traditional Chinese Medicine, Shanghai, China

**Keywords:** Mortality, Disability, Grip strength, Gait speed, Chair stands test, Older adults

## Abstract

**Background:**

The link between low grip strength, diminished physical performance, and adverse health outcomes in older adults has been well-established. However, the impact of older adults who cannot complete these tests on disability and mortality rates remains unexplored without longitudinal study.

**Methods:**

We collected data from the China Health and Retirement Longitudinal Study (CHARLS). Participants aged 60–101 were enrolled at baseline. We analyzed the prevalence of populations unable to complete handgrip strength (HGS), gait speed (GS), and five times chair stand test (FTCST). Completing risk models were used to estimate the risk of mortality and disability over seven years.

**Results:**

A total of 3,768 participants were included in the analysis. The percentage of older adults unable to complete the GS and FTCST tests increased notably with age, from 2.68 to 8.90% and 2.60–20.42%, respectively. The proportion of older people unable to perform the HGS was relatively stable, ranging from 1.40 to 3.66%. Compared to older adults who can complete these tests, those who cannot perform FTCST face a significantly higher risk of mortality, with 49.1% higher risk [hazard ratio (HR) = 1.491, 95% CI = 1.156, 1.922; subdistribution hazard ratio (SHR) = 1.491, 95%CI = 1.135,1.958)]. Participants who were unable to complete the GS test had a higher risk of developing ADL disability, regardless of whether they were compared to the lowest-performing group (HR = 1.411, 95%CI = 1.037,1.920; SHR = 1.356, 95%CI = 1.030,1.785) or those who can complete the GS (HR = 1.727, 95%CI = 1.302,2.292; SHR = 1.541, 95%CI = 1.196,1.986). No statistically significant difference in the risk of developing ADL disability among older adults who were unable to complete the HGS test compared with either the poorest performing group (HR = 0.982, 95% CI = 0.578, 1.666; SHR = 1.025, 95% CI = 0.639, 1.642) or those who were able to complete the HGS test (HR = 1.008, 95% CI = 0.601, 1.688; SHR = 0.981, 95% CI = 0.619, 1.553). The risk of all-cause mortality was not significantly different for older adults who were unable to complete the HGS test compared to those with the worst performance (HR = 1.196, 95%CI = 0.709–2.020; SHR = 1.196, 95%CI = 0.674, 2.124) or those who were able to complete the test (HR = 1.462, 95%CI = 0.872–2.450; SHR = 1.462, 95%CI = 0.821,2.605).

**Conclusion:**

The risks of adverse events faced by older adults unable to complete the tests vary, indicating the necessity for future research to conduct separate analyses on this high-risk population.

**Supplementary Information:**

The online version contains supplementary material available at 10.1186/s12889-024-18258-7.

## Introduction

Previous meta-analyses studies have demonstrated that a decline in grip strength or physical performance among older adults is associated with adverse health outcomes, including disability and mortality [1–4]. Although these objective physical measures are commonly employed as proxies for assessing individual health status, it is essential to acknowledge that the feasibility of each instrument can vary, particularly for older individuals who may face challenges in completing these tests [4].

Limited information exists regarding the prevalence of older adults unable to complete grip strength, gait speed, and chair stand tests, as well as the potential negative impact of these populations on adverse outcomes. Only a handful of studies have delved into this area [5–9]. Notably, one study disclosed a significant increase in the percentage of individuals unable to perform the five times chair stand test, rising from 34.4% in those aged 65–69 to 89.7% in those aged 90 and above [9]. Furthermore, older individuals unable to complete the repeated chair stand and grip strength tests were associated with a heightened risk of all-cause mortality [7]. As such, it remains unclear how the prevalence of older adults who cannot conduct these three objective physical measurements and their association with disability and mortality in China, if any. Addressing this knowledge gap can aid healthcare practitioners in more comprehensively assessing patient prognosis and assist policymakers in understanding the potential impact of adverse health events in older adults facing challenges in completing these tests, especially in regions with disparities in healthcare resource allocation.

Therefore, this study aimed to calculate the prevalence of older people without the ability to accomplish grip strength, gait speed, and chair stand test and to investigate the longitudinal association of these populations with activities of daily living (ADL) disability and all-cause mortality.

## Methods

### Study populations

The data utilized in this study were sourced from the China Health and Retirement Longitudinal Study (CHARLS), an ongoing longitudinal investigation with a nationally representative sample of middle-aged and older individuals residing in Chinese communities. The initial sample encompassed 10,257 households within 450 villages and urban communities across 28 provinces, broadly capturing China’s annual older population. Detailed information regarding the CHARLS study has been previously published [10]. In brief, the baseline survey began in 2011 (Wave 1) with 17,708 participants, and subsequent follow-ups were conducted from 2013 (Wave 2) to 2018 (Wave 4). For this study, data analysis focused on individuals aged 60 years and above who had comprehensive grip strength, gait speed, and the five times sit-to-stand test results at the initial wave. Exclusion criteria included: (1) individuals under the age of 60; (2) missing data, including age, activities of daily living, grip strength, gait speed, and five-times chair stand test; (3) self-reported emotional, nervous, or psychiatric problems; (4) self-reported memory-related conditions; (5) reported experiencing difficulty in any of the four activities of daily living at baseline survey. The flowchart outlining the inclusion and exclusion criteria can be found in Figure S1. Ethics approval for the data collection was obtained by the original researchers of CHARLS from the Ethical Review Committee of Peking University (IRB00001052–11,015).

### Assessment of grip strength and physical measures

For each measure, trained staff recorded if a respondent was unable to complete the test, including surgery, swelling, or other health reasons, as well as those who had limited understanding of the instructions of each test and those who tried but were unable to finish the test (i.e., those who cannot compete for the total five-time sit-to-stand test). Evidence suggests these physical measures are reliable and valid [11–13].

1. Handgrip strength (HGS) was assessed using a mechanical dynamometer, measured in kilograms. Participants held the dynamometer with one hand, maintaining a 90° elbow flexion while seated or standing, and exerted maximum force by squeezing the dynamometer for a brief duration. Two readings were recorded for each hand, and the highest value was selected to represent the individual’s grip strength.
2. The gait speed (GS) was expressed in meters per second. For two consecutive trials, participants were directed to walk 2.5 m along an uncarpeted 4-meter walking course, adhering to their habitual pace. The use of walking aids was permitted during the assessments. Stopwatch timing was employed to measure the walking duration, and the average value derived from the repeated measurements was employed for the analysis.
3. The five-times chair stand test (FTCST), also known as the repeated chair stands, entailed timing the seconds for individuals to move from a seated position to a fully upright standing position, then returning to a seated position, repeating this sequence five times consecutively. During the test, participants kept their arms folded across their chests and refrained from using their arms to aid in pushing off. This trial was administered only once.

### Assessment of all-cause mortality and disability

Among the three survey waves, only Wave 2 (2013) included the precise date of death, whereas Wave 3 (2015) and 4 (2018) solely gathered information on the mortality status. Consequently, to determine the survival time, the midpoint between the initial visit date and the date when the respondent’s death was documented was computed [14]. The assessment of ADL disability was primarily based on the self-report of at least one basic ADL difficulty using the Katz ADL scale, encompassing daily self-care tasks, including bathing, dressing, eating, getting in/out of bed, using the toilet, and controlling urination [15].

### Covariates

Potential confounding variables associated with disability and mortality were identified based on existing literature [1–9, 14]. This included demographic characteristics such as age, gender, educational attainment, and urban/rural. Lifestyle factors, including physical activity levels, smoking behavior, and alcohol consumption, were also considered. Health conditions, encompassing BMI, self-reported presence of conditions, cognitive function, and depressive symptoms, were included as well.

### Demographic characteristics

To facilitate cross-country education level comparisons, we classified education into three scales. Participants reporting an education level of “middle school” and below were categorized as “less than lower secondary education.” Those with an education level of “High School” or “Vocational School” were classified as “upper secondary & vocational training.” Participants who had obtained a bachelor’s degree or above were categorized under “Tertiary education.” Additionally, respondents were categorized as urban or rural based on their place of residence, following the classification provided by the National Bureau of Statistics China.

### Lifestyle factors

Respondents in CHARLS were queried their weekly physical activity (PA) status, encompassing intensity (vigorous PA, moderate PA, and walking), duration, and frequency. Following the IPAQ protocol and the guidance from a prior study based on CHARLS [16], we computed the total volume of PA in MET-minutes/week, categorizing it into three levels: low, moderate, and high PA. The total volume of PA was determined by multiplying the average duration per day (minutes/day) by frequency (days). Notably, respondents were prompted to provide a range of duration time rather than the precise minutes of each PA per day, requiring the calculation of an average duration time value. Smoking and drinking status were categorized as never, former, and current.

### Health conditions

Body mass index (BMI) was calculated by dividing weight in kilograms by the square of height in meters. In alignment with the WHO recommendation for the Asian and South Asian population, BMI was classified into four categories: underweight (BMI < 18.5), normal (18.5 ≤ BMI < 23), and overweight (23 ≤ BMI < 25), obese (BMI ≥ 25). The self-report of physician diagnosis chronic conditions, including hypertension, cancer, diabetes, chronic lung disease (chronic bronchitis or emphysema), and cardiovascular diseases (heart disease and stroke), was dichotomized into yes/no categories.

Cognitive status was assessed through a total score of 21, incorporating Telephone Interview of Cognitive Status (TICS-10), episodic memory, and visual-spatial ability test. TICS-10, a validated method with a total score of 10, involved participants identifying today’s date (day, month, and year), the day of the week, and the current season. Additionally, participants performed seven subtractions of numbers from 100 five times. Episodic memory was evaluated by recalling a 10-word list, with participants earning one point for each correct recall. Visual-spatial ability was tested by assessing whether the respondent could accurately copy an assigned picture, with participants receiving one point for successful reproduction.

Depression status was evaluated using a validated 10-item Center for Epidemiologic Studies Depression Scale (CES-D). Participants rated the frequently of experiencing depressive symptoms over the past week, with each item scored from 0 to 3. After reversing two positive items (feeling hopeful and happy), a total score was calculated by summing all ten-item scores. A total score exceeding 12 indicates the presence of depression [17].

### Statistical analysis

A descriptive analysis was conducted to present the baseline characteristics of the sample. The prevalence of grip strength, gait speed, and chair stand test categories was illustrated, stratified by age and sex groups, with individuals aged 80 and above grouped together. In this study, we employed competing risk analysis, specifically the Fine-Gray model, to examine incident ADL disability, with mortality considered the competing event [18]. This analytical approach takes into account the possibility that participants may pass away before developing ADL disability. To provide a more comprehensive understanding of the relationship between covariates and the outcome of interest, accounting for competing events, we simultaneously fitted both cause-specific hazard models and Fine-Gray sub-distribution hazard models simultaneously [19]. The models were adjusted age, sex, body size, sociodemographic characteristics, lifestyle factors, and health conditions. The proportional-hazards assumption was evaluated using Schoenfeld residuals, revealing no indications of violation. Additionally, a likelihood ratio test was conducted to assess the interaction effect of sex.

To enhance statistical power and address potential bias stemming from missing covariates, we employed imputation techniques to fill in missing values in a sample of participants with complete data on objective measures and all-cause mortality. The covariates with missing values included hypertension (0.3%), diabetes (3.3%), cancer (0.1%), lung disease (0.1%), cardiovascular diseases (0.7%), height (0.6%), physical activity levels (27.9%), smoking status (0.6%), depression status (1.7%), and cognitive status (15.1%). Notably, a significant proportion of missing values was observed for physical activity levels, primarily because only about two-fifths of all CHARLS participants were randomly selected to respond to the items. It was reasonable to assume these missing values to be missing at random. Consequently, we performed multiple imputations using chained equations, generating five datasets that were subsequently pooled together for analysis in cause-specific and Fine-Gray sub-distribution hazard models.

*P* values ≤ 0.05 were considered statistical significance. All analyses were conducted with Stata 17.0 (Stata Corporation, College Station, TX).

## Results

### Sample characteristics

Out of the initial total of 4,166 participants in the study, 398 individuals could not be traced, and 633 individuals passed away during the seven-year follow-up, leaving 3,768 participants for analysis. The incidence rate of ADL disability was 33.02 (95%CI = 30.80–35.40) per 1000 person-years, and the incidence rate of mortality was 26.29 (95%CI = 24.32–28.42) per 1000 person-years. The sample was predominantly composed of participants with low levels of education (96.85%), residing in rural areas (65.91%), and engaging in low levels of physical activity (65.98%). Among the participants, men accounted for 54.16% of the sample. Women had a higher rate of being overweight (21.38%) and obese (29.68%). Furthermore, women were more likely to be non-smokers and drinkers, while men had higher rates of current smoking and alcohol consumption. Hypertension was the most prevalent among the self-reported chronic diseases investigated (31.35%), followed by cardiovascular disease (15.14%) and chronic lung disease (12.48%), as shown in Table 1.

**Table 1:** Characteristics of study sample at baseline stratified by genders (*n* = 3768)

Characteristics [*n* (%) unless shown otherwise]	*N*	Men(*n* = 1992)	Women(*n* = 1776)	*P* value
Age (years), mean (SD)	3768	67.33 ± 6.02	67.69 ± 6.69	0.086
Education	3768			< 0.001
Tertiary	50	39 (1.96)	11 (0.62)	
Upper secondary & vocational training	156	119 (5.97)	37 (2.08)	
Less than lower secondary	3562	1834 (92.07)	1728 (97.30)	
Residence	3768			0.032
Urban community	1344	679 (34.09)	665 (37.44)	
Rural Village	2424	1313 (65.91)	1111 (62.56)	
Height (m), mean (SD)	3742	1.62 ± 0.08	1.50 ± 0.08	< 0.001
BMI (kg/m2), median (P_25_-P_75_)	3729	21.97(19.91–24.31)	23.09(20.54–25.60)	< 0.001
BMI	3729			< 0.001
Underweight	383	192 (9.75)	191 (10.86)	
Normal	1690	1020 (51.78)	670 (38.09)	
Overweight	732	356 (18.07)	376 (21.38)	
Obesity	924	402 (20.41)	522 (29.68)	
Smoking status	3749			< 0.001
Never	2106	528 (26.71)	1578 (89.05)	
Former	422	373 (18.87)	49 (2.77)	
Current	1221	1076 (54.43)	145 (8.18)	
Drinking status	3768			< 0.001
Never	2151	678 (34.04)	1473 (82.94)	
Former	622	479 (24.05)	143 (8.05)	
Current	995	835 (41.92)	160 (9.01)	
Physical activity	2719			< 0.001
Low	1794	931 (65.01)	863 (67.06)	
Moderate	683	342 (23.88)	341 (26.50)	
High	242	159 (11.10)	83 (6.45)	
Hypertension	3757			< 0.001
No	2579	1431 (72.02)	1148 (64.86)	
Yes	1178	556 (27.98)	622 (35.14)	
Diabetes	3647			0.011
No	3513	1880 (94.47)	1633 (92.42)	
Yes	244	110 (5.53)	134 (7.58)	
Cancer	3766			0.347
No	3735	1973 (99.05)	1762 (99.32)	
Yes	31	19 (0.95)	12 (0.68)	
Chronic Lung disease	3765			< 0.001
No	3295	1693 (85.08)	1602 (90.25)	
Yes	470	297 (14.92)	173 (9.75)	
Cardiovascular disease	3745			0.954
No	3178	1682 (84.52)	1496 (84.28)	
Yes	567	297 (14.92)	270 (15.21)	
Cognition function score	3199	7.40 ± 3.22	6.77 ± 3.49	< 0.001
CES-D score, mean (SD)	3707	7.09 ± 5.42	8.71 ± 6.18	< 0.001
CES-D score	3707			< 0.001
≥ 12	890	378 (19.26)	512 (29.36)	
< 12	2817	1585 (80.74)	1232 (70.64)	
Grip strength	3768			0.403
Able	3717	1968 (98.80)	1749 (98.48)	
Unable	51	24 (1.20)	27 (1.52)	
Grip strength (kg), mean (SD)	3768	35.62 ± 8.37	24.19 ± 6.93	< 0.001
Gai speed	3768			0.052
Able	3593	1912 (95.98)	1681 (94.65)	
Unable	175	80 (4.02)	95 (5.35)	
Gai speed (m/s), mean (SD)	3768	0.67 ± 0.21	0.61 ± 0.21	< 0.001
Five times sit to stand	3768			< 0.001
Able	3543	1904 (95.58)	1639 (92.29)	
Unable	225	88 (4.42)	137 (7.71)	
Five times sit to stand (s), mean (SD)	3768	10.68 ± 3.70	12.21 ± 5.13	< 0.001

Note: For categorical variables, the potential differences among groups were employed by one-way analysis of variance (ANOVA) (normal distribution) or Kruskal Wallis rank sum test (skewed distribution). For the categorical variables, we employed the chi-squared test to identify any significant differences across various groups. BMI, body mass index; CES-D, Center for Epidemiologic Studies Depression Scale; SD, standard deviation

### Prevalence of populations unable to complete tests

**Fig. 1:**
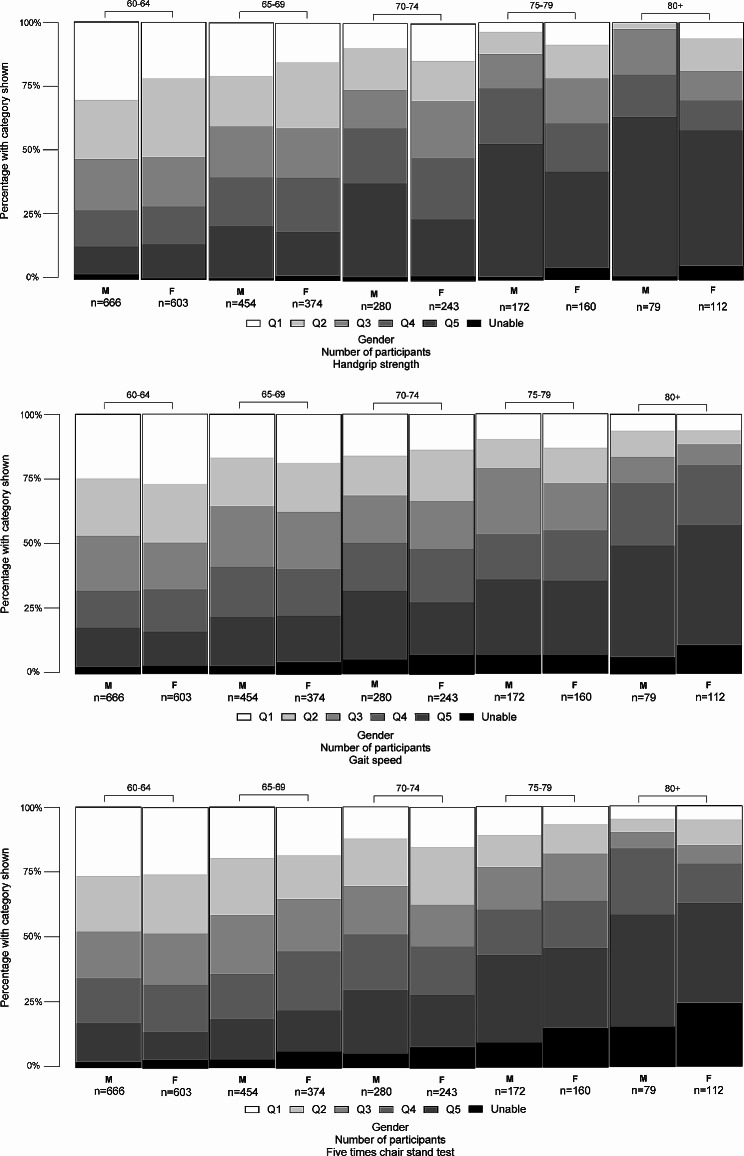
Bar graphs depicting the three assessments, stratified by gender and age

The decline in the performance of older people in HGS, as well as FTCST and GS with age was evident, as shown in Fig. 1. With advancing age, there was a significant increase in the percentage of people unable to perform the FTCST and GS, rising from 2.60 to 20.42% and 2.68–8.90%, respectively. However, the proportion of individuals unable to perform the HGS remained relatively stable, with rates of 1.10% in the 60–64 age group and 3.66% in the 80 + age group. Furthermore, the proportion of participants classified in the lowest fifth for handgrip strength was relatively low at 11.98% in the 60–64 age group but significantly surged to 56.54% in those aged 80 and above. A similar trend was observed for the FTCST and GS, with the proportion of performers in the lowest fifth category rising from 12.84 to 40.31% and 14.26–45.03%, respectively.

The number of individuals who could not finish the FTCST (*n* = 225) was significantly greater than those who faced difficulties in completing the HGS (*n* = 51) and GS (*n* = 175). Most of the reasons for not being able to complete the tests are related to health issues (98.04% for HGS, 83.43% for GS, 88.89% for FTCST). Among these three tests, a significant proportion of individuals (26.67%) attempted but could not complete the FTCST compared to the other two tests (1.96% for HGS and 4.57% for GS).

### Cause-specific hazard model

There was no difference between sex and all three measures (all *P* > 0.05 from each test-sex interaction), prompting the inclusion of two groups to maximize power in subsequent models. As shown in Table 2, compared to the best-performing group, an inability to complete the GS and FTCST was independently associated with 96.9% (HR = 1.969, 95% CI = 1.412–2.745) and 74.46% (HR = 1.746, 95% CI = 1.278–2.386) increased risk of ADL disability, respectively. Although participants unable to complete the HGS test had an increased risk of disability, this difference did not reach statistical significance.

In the context of the competing event of death, among participants who were alive and free from ADL disability, older adults unable to complete the GS test did not show a significant association with the risk of death (HR = 1.355, 95% CI = 0.926–1.986). However, those who were unable to complete the HGS and FTCST demostrated an increased risk of death by 87.6% (HR = 1.876, 95% CI = 1.057–3.329) and 122% (HR = 2.221, 95% CI = 1.559–3.164), respectively, as detailed in Table 3.

Participants who were unable to complete the GS test faced a higher risk of developing ADL disability, whether compared to the lowest-performing group (HR = 1.411, 95% CI = 1.037, 1.920) or those who could complete the GS (HR = 1.727, 95% CI = 1.302, 2.292). Furthermore, older adults unable to complete the FTCST, when compared to those who could, experienced a 45.1% increased risk (HR = 1.451, 95% CI = 1.127–1.869) for ADL disability and a 49.1% increased risk (HR = 1.491, 95% CI = 1.156–1.922) for mortality. Failure to complete the HGS test put participants at a heightened risk of mortality, even when compared to those with the worst performance (HR = 1.196, 95%CI = 0.709–2.020) or those who were able to finish the test (HR = 1.462, 95%CI = 0.872–2.450). However, this risk did not reach statistical significance. The risk of ADL disability showed no significant difference among older adults who were unable to complete the HGS test, those with the poorest performance level (HR = 0.982, 95%CI = 0.578–1.666), and those who were able to complete the test (HR = 1.008, 95%CI = 0.601–1.688), as presented in Table 4.

### Fine-gray subdistribution hazard model

In the subdistribution hazard model for incident ADL disability with mortality as the competing event, the results align with those obtained from the cause-specific hazard model. Older adults who could not complete the Grip Strength (GS) and Five Times Chair Stand Test (FTCST) were independently associated with a 78.7% (SHR = 1.787, 95% CI = 1.319–2.420) and a 42.8% (SHR = 1.428, 95% CI = 1.071–1.904) increased risk of ADL disability, respectively (see Table 2).

In the Fine-Gray hazard model for incident mortality with ADL disability as the competing event, older adults unable to complete the GS test did not exhibit a significant association with the risk of death (SHR = 1.356, 95% CI = 0.922–1.993). However, those who could not conduct the Hand Grip Strength (HGS) and FTCST tests showed an increased risk of death by 87.6% (SHR = 1.876, 95% CI = 1.013–3.474) and 122% (SHR = 2.221, 95% CI = 1.545–3.193), as displayed in Table 3.

For participants who could not complete the GS test, they faced a higher risk of developing ADL disability, whether compared to the lowest-performing group (SHR = 1.356, 95% CI = 1.030–1.785) or those who could complete the GS (SHR = 1.541, 95% CI = 1.196–1.986). Compared to those who could, older adults unable to complete the FTCST experienced a 49.1% increased risk of mortality (SHR = 1.491, 95% CI = 1.135–1.958). Although participants who could not complete the FTCST demonstrated an increased risk of disability, it did not reach statistical significance (SHR = 1.225, 95% CI = 0.977–1.534). The all-cause mortality risk for older adults unable to complete the HGS test was not significantly different from those with the poorest performance level (SHR = 1.196, 95%CI = 0.674–2.124) or those able to complete the HGS test (SHR = 1.462, 95%CI = 0.821–2.605). Similarly, this group of older adults, unable to complete the HGS test, also showed no significant difference in the risk of ADL disability compared to those with the poorest performance (SHR = 1.025, 95%CI = 0.639–1.642) or those able to complete the HGS test (SHR = 0.981, 95%CI = 0.619–1.553), as indicated in Table 4.

**Table 2:** Competing risk analysis of incident ADL disability, with mortality as the competing event (*n* = 3768, deaths = 633, disability = 795)

	*N* (events/groups)	Cause-specific hazard model (HR,95%CI)		Sub-distribution hazard model (SHR, 95%CI)	
		Adjusted for age and sex	Full-adjusted ^b^	Adjusted for age and sex	Full-adjusted ^b^
Handgrip strength (kg) ^a^					
Unable	15/36	1.457(0.849,2.500)	1.067(0.617,1.847)	1.251(0.759,2.062)	1.015(0.622,1.658)
Q5	199/621	1.322(1.043,1.674)	1.087(0.847,1.396)	1.161(0.927,1.454)	0.991(0.787,1.247)
Q4	152/559	1.212(0.950,1.546)	1.123(0.874,1.444)	1.169(0.930,1.469)	1.091(0.864,1.378)
Q3	149/622	1.097(0.861,1.399)	0.991(0.773,1.270)	1.124(0.896,1.410)	1.041(0.830,1.306)
Q2	161/699	1.120(0.883,1.420)	1.057(0.831,1.343)	1.120(0.896,1.399)	1.078(0.865,1.344)
Q1	119/598	Ref.	Ref.	Ref.	Ref.
Gait speed (m/s) ^a^					
Unable	54/134	2.032(1.462,2.824)	1.969(1.412,2.745)	1.889(1.400,2.549)	1.787(1.319,2.420)
Q5	187/571	1.616(1.272,2.052)	1.395(1.095,1.778)	1.486(1.183,1.864)	1.318(1.048,1.657)
Q4	151/568	1.311(1.024,1.677)	1.113(0.867,1.428)	1.304(1.036,1.640)	1.162(0.922,1.464)
Q3	172/655	1.359(1.070,1.726)	1.225(0.964,1.558)	1.407(1.124,1.761)	1.297(1.038,1.621)
Q2	119/588	0.982(0.759,1.272)	0.887(0.684,1.151)	0.989(0.776,1.260)	0.920(0.722,1.172)
Q1	112/619	Ref.	Ref.	Ref.	Ref.
Five-times chair stand test (s) ^a^					
Unable	70/152	1.965(1.444,2.674)	1.746(1.278,2.386)	1.638(1.232,2.178)	1.428(1.071,1.904)
Q5	181/525	1.581(1.242,2.013)	1.333(1.041,1.707)	1.434(1.141,1.804)	1.222(0.966,1.545)
Q4	149/571	1.414(1.104,1.811)	1.220(0.949,1.569)	1.307(1.037,1.648)	1.166(0.923,1.472)
Q3	141/612	1.246(0.972,1.599)	1.119(0.870,1.439)	1.228(0.972,1.522)	1.131(0.894,1.430)
Q2	143/625	1.316(1.027,1.687)	1.228(0.957,1.576)	1.291(1.023,1.630)	1.228(0.975,1.548)
Q1	111/650	Ref.	Ref.	Ref.	Ref.

*Note: a.* Physical measures grouped into six categories: Unable; Q5(Quintile5, lowest performance); Q4; Q3; Q2; Q1(Quintile1, highest performance)

b. Full adjusted for age and sex plus height and BMI, sociodemographic factors (education and residence), lifestyles (physical activity levels, smoking behavior, and alcohol consumption), and medical conditions (hypertension, diabetes, cancer, cardiovascular disease, chronic lung disease, depression status, and cognitive status). Ref, reference. ADL, activities of daily living. HR, hazard ratio. CI, confidence intervals. *N*, numbers

**Table 3:** Competing risk analysis of incident mortality, with ADL disability as the competing event (*n* = 3768, deaths = 633, disability = 795)

	*N* (events/groups)	Cause-specific hazard model (HR, 95%CI)		Sub-distribution hazard model (SHR, 95%CI)	
		Adjusted for age and sex	Full-adjusted ^b^	Adjusted for age and sex	Full-adjusted ^b^
Handgrip strength (kg) ^a^					
Unable	15/51	2.081(1.181,3.665)	1.876(1.057,3.329)	2.080(1.147,3.775)	1.876(1.013,3.474)
Q5	249/870	1.765(1.332,2.340)	1.568(1.166,2.107)	1.765(1.337,2.331)	1.568(1.169,2.103)
Q4	127/686	1.390(1.031,1.875)	1.300(0.956,1.767)	1.390(1.033,1.870)	1.300(0.959,1.762)
Q3	89/711	1.016(0.739,1.396)	0.976(0.707,1.348)	1.016(0.742,1.392)	0.976(0.709,1.343)
Q2	85/784	1.011(0.734,1.392)	0.998(0.723,1.378)	1.011(0.735,1.390)	0.998(0.724,1.377)
Q1	68/666	Ref.	Ref.	Ref.	Ref.
Gait speed (m/s) ^a^					
Unable	41/175	1.497(1.025,2.186)	1.356(0.926,1.986)	1.497(1.022,2.192)	1.356(0.922,1.993)
Q5	190/761	1.512(1.160,1.970)	1.317(1.006,1.723)	1.512(1.155,1.977)	1.317(1.003,1.729)
Q4	122/690	1.184(0.893,1.569)	1.046(0.787,1.390)	1.184(0.891,1.573)	1.046(0.786,1.391)
Q3	100/750	0.981(0.732,1.314)	0.882(0.658,1.185)	0.981(0.731,1.317)	0.883(0.657,1.186)
Q2	98/686	1.218(0.908,1.633)	1.108(0.825,1.488)	1.218(0.906,1.637)	1.108(0.825,1.488)
Q1	82/701	Ref.	Ref.	Ref.	Ref.
Five-times chair stand test (s)^a^					
Unable	73/225	2.440(1.719,3.463)	2.221(1.559,3.164)	2.440(1.705,3.493)	2.221(1.545,3.193)
Q5	179/704	2.014(1.503,2.700)	1.819(1.349,2.453)	2.014(1.513,2.681)	1.819(1.357,2.439)
Q4	135/706	1.768(1.309,2.386)	1.589(1.173,2.152)	1.768(1.310,2.384)	1.589(1.173,2.154)
Q3	96/708	1.362(0.992,1.870)	1.302(0.946,1.791)	1.362(0.995,1.864)	1.302(0.947,1.790)
Q2	86/711	1.248(0.902,1.725)	1.198(0.865,1.658)	1.248(0.904,1.722)	1.198(0.867,1.655)
Q1	64/714	Ref.	Ref.	Ref.	Ref.

*Note: a.* Physical measures grouped into six categories: Unable; Q5(Quintile5, lowest performance); Q4; Q3; Q2; Q1(Quintile1, highest performance)

b. Full adjusted for age and sex plus height and BMI, sociodemographic factors (education and residence), lifestyles (physical activity levels, smoking behavior, and alcohol consumption), and medical conditions (hypertension, diabetes, cancer, cardiovascular disease, chronic lung disease, depression status, and cognitive status). Ref, reference. ADL, activities of daily living. HR, hazard ratio. CI, confidence intervals. *N*, numbers

**Table 4:** Associations between inability to complete and death or disability with full adjusted, compared to the worst-performing and able-to-complete groups

	All-cause mortality			ADL disability		
	*N* (events/groups)	Cause-specific hazard model (HR,95%CI)	Sub-distribution hazard model (SHR, 95%CI)	*N* (events/groups)	Cause-specific hazard model (HR,95%CI)	Sub-distribution hazard model (SHR, 95%CI)
Handgrip strength (kg)^a^						
Unable	15/51	1.196(0.709,2.020)	1.196(0.674,2.124)	15/36	0.982(0.578,1.666)	1.025(0.639,1.642)
Q5	249/870	Ref	Ref	199/621	Ref	Ref
Unable	15/51	1.462(0.872,2.450)	1.462(0.821,2.605)	15/36	1.008(0.601,1.688)	0.981(0.619,1.553)
Able	618/3717	Ref	Ref	780/3099	Ref	Ref
Gait speed (m/s) ^a^						
Unable	41/175	1.030(0.732,1.448)	1.030(0.728,1.457)	54/134	1.411(1.037,1.920)	1.356(1.030,1.785)
Q5	190/761	Ref	Ref	187/571	Ref	Ref
Unable	41/175	1.238(0.898,1.706)	1.238(0.894,1.715)	54/134	1.727(1.302,2.292)	1.541(1.196,1.986)
Able	592/3593	Ref	Ref	741/3001	Ref	Ref
Five times sit-to-stand (s)^a^						
Unable	73/225	1.221(0.926,1.610)	1.221(0.915,1.629)	70/152	1.310(0.990,1.733)	1.169(0.913,1.497)
Q5	179/704	Ref	Ref	181/525	Ref	Ref
Unable	73/225	1.491(1.156,1.922)	1.491(1.135,1.958)	70/152	1.451(1.127,1.869)	1.225(0.977,1.534)
Able	560/3543	Ref	Ref	725/2983	Ref	Ref

*Note: a.* Physical measures grouped into two categories: Unable, for individuals unable to complete the test; Q5(Quintile5, lowest performance); Able, for individuals able to complete the test

b. Full adjusted for age and sex plus height and BMI, sociodemographic factors (education and residence), lifestyles (physical activity levels, smoking behavior, and alcohol consumption), and medical conditions (hypertension, diabetes, cancer, cardiovascular disease, chronic lung disease, depression status, and cognitive status). Ref, reference. ADL, activities of daily living. HR, hazard ratio. CI, confidence intervals. *N*, numbers

## Discussion

We found that a proportion of individuals aged 60 and above face challenges in completing physical performance tests such as gait speed and chair stand tests. This issue became more prevalent with advancing age, while this trend was not observed in grip strength. Our study extended previous research conducted in the United Kingdom [9] (among individuals aged 65 to 90 and older) and the United States [5] (among individuals aged 71 to 80 and older) by not only focusing on individuals aged 60 to 80 and above in the Chinese population but also, for the first time, examining older adults facing challenges in completing grip strength assessments.

We further investigated the association between this population and subsequent adverse health events. The previous study only examined the higher hazard ratios for all-cause mortality in people unable to accomplish the grip strength, standing balance, and chair stand test compared to those in the highest quintile [7]. Instead, we focused on whether participants facing challenges or performing poorly in these tests had higher rates of ADL disability and mortality. Our findings revealed that older adults unable to perform GS and FTCST had varied risks for adverse event outcomes. While those unable to complete GS or FTCST did not differ in the risk of death compared to the worst-performing group, older adults unable to perform the gait test faced a higher risk of ADL disability than the worst-performing group. These results emphasize the importance of considering distinct groups (unable vs. poorly performing) when assessing specific events, particularly for gait speed.

Our research findings highlight the importance of grip strength as a crucial screening tool in clinical settings, specifically for conditions like frailty [20] and sarcopenia [21–23], particularly among individuals aged 80 and above. Given that a significant portion of this population may encounter difficulties in completing tests involving gait speed and chair stand tests, grip strength emerges as a valuable and simple biomarker with predictive power for subsequent health conditions across the lifespan [2, 7, 24–27]. It adds value to traditional risk factors in the predicting conditions such as diabetes [28] and functional disability [29, 30]. Notably, our study found that older adults unable to undergo HGS testing did not show statistically significant differences in estimated mortality and disability risk compared to those in the lowest quintile or those able to undergo HGS testing. The relatively wide confidence intervals for these risk estimates indicate the need for further research and validation due to small sample size.

Our study identified a floor effect in the chair stand test for older individuals, with some unable to complete five attempts, resulting in an absence of a score for evaluating the severity of their performance levels. As a potential alternative to the five-times chair stands test, the 30-second chair stand test is deemed more appropriate for assessing functional capacity in older adults [31]. However, additional research is necessary for populations encountering challenges with the five times chair stand test to assess the extent of decline in assessment when transitioning to the 30-second chair stand test and its implications for the adverse outcomes.

The study also has some limitations. First, our findings were based on older people, which should not be extrapolated to younger people. However, a previous study suggested that the inability to perform grip strength, repeated chair stands, and standing balance have also increased the risk of future mortality at age 53 [7]. Second, we cannot apply subgroup analysis to examine the association between older people unable to accomplish these tests and specific causes of death, such as cancer, due to limitations of the number from self-reported conditions. Finally, given the wide confidence intervals in the risk estimates for adverse events among older people unable to complete grip strength tests, further research is warranted for validation.

## Conclusion

Using a representative sample of older adults from China, our research revealed that the proportion of older adults unable to perform the chair stand test and gait speed assessments tended to increase with age. In contrast, the proportion of older people incapable of completing grip strength test remained relatively low and had a stable trend with advancing age. The risks of adverse events among older adults unable to complete the tests vary in comparison to those who had the poorest performance, suggesting the need for independent analyses of this high-risk group, rather than merging them with individuals displaying the lowest performance, to better understand and address the distinct characteristics associated with heightened risk in this subset of the older populations.

### Electronic supplementary material

Below is the link to the electronic supplementary material.

Supplementary Material 1

## Data Availability

The datasets generated and analyzed during the current study are available in the CHARLS database, publicly available on the CHARLS website (http://charls.pku.edu.cn/).
