# Supplementary material for: Mortality and disability risk among older adults unable to complete grip strength and physical performance tests: a population-based cohort study from China
Source: BMC Public Health. 2024 Mar 13;24:797. doi: 10.1186/s12889-024-18258-7 (PMC10938679; doi:10.1186/s12889-024-18258-7)
Supplement: Supplementary file 1 — Supplementary Material 1 [file 12889_2024_18258_MOESM1_ESM.docx]

Mortality and disability risk among older adults unable to complete grip strength and physical performance tests: a population-based cohort study from China

Supporting information

1. Supplementary Figure S1: flowchart of the inclusion and exclusion criteria

**
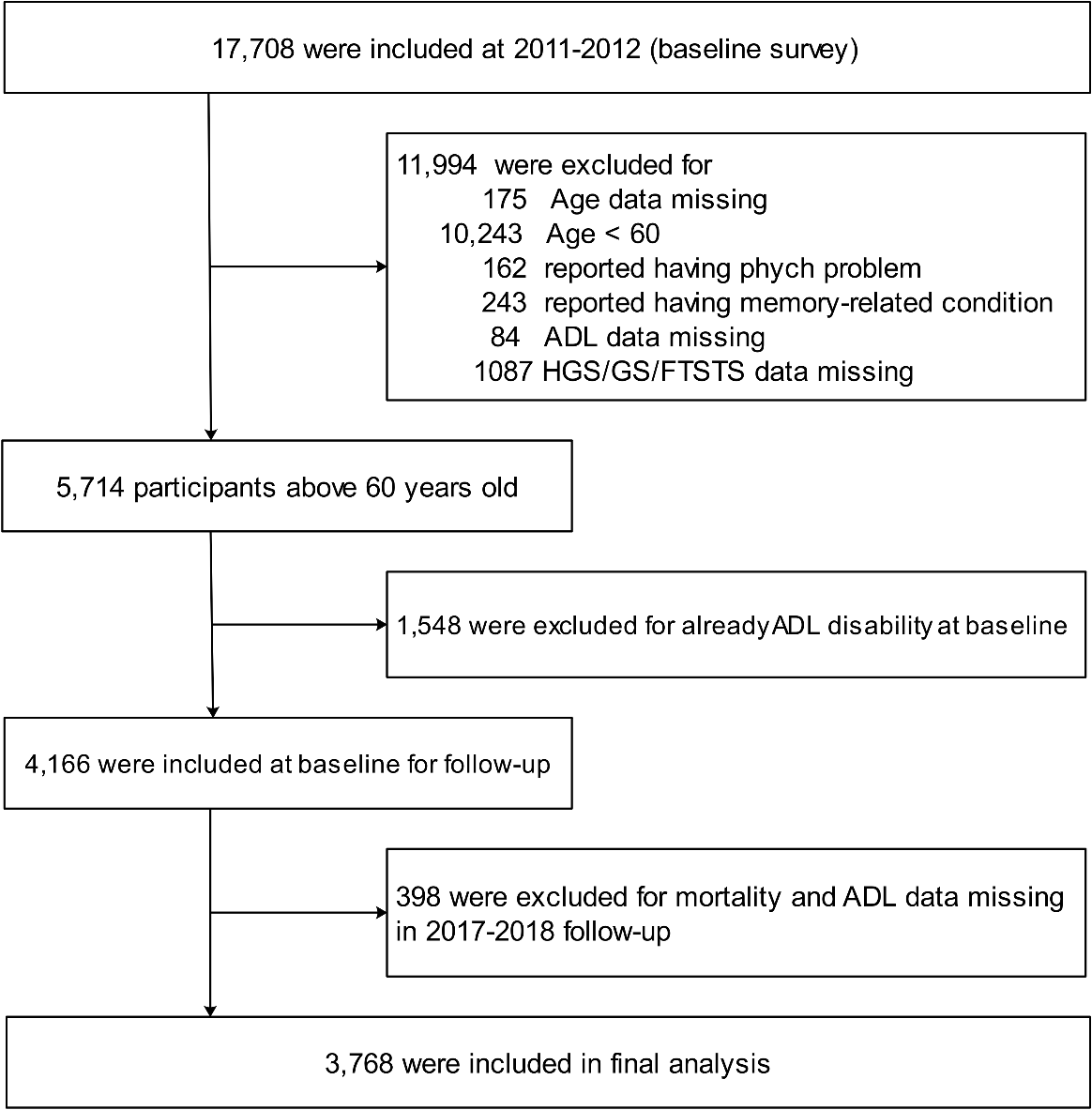
**
